# Circulation of human respiratory syncytial virus and new ON1 genotype in northern Viet Nam, 2017–2020

**DOI:** 10.5365/wpsar.2023.14.4.990

**Published:** 2023-10-16

**Authors:** Ung Thi Hong Trang, Hoang Vu Mai Phuong, Nguyen Huy Hoang, Nguyen Vu Son, Le Thi Thanh, Nguyen Le Khanh Hang, Vuong Duc Cuong, Tran Thi Thu Huong, Nguyen Thi Hien, Nguyen Phuong Anh, Le Quynh Mai

**Affiliations:** a Graduate University of Science and Technology, Vietnam Academy of Science and Technology, Hanoi, Viet Nam.; b National Institute of Hygiene and Epidemiology, Hanoi, Viet Nam.; c Institute of Genome Research, Vietnam Academy of Science and Technology, Hanoi, Viet Nam.

## Abstract

**Objective:**

Human respiratory syncytial virus (RSV) is a primary cause of paediatric severe acute respiratory infection (SARI) worldwide, especially in developing countries. We investigated the genetic characteristics of RSV in northern Viet Nam to determine the prevalence and distribution of subtypes as well as the diversity and transmission patterns of genotypes.

**Methods:**

In two facilities, from January 2017 to December 2020, 1563 clinical specimens were collected from paediatric patients hospitalized with SARI and tested for RSV. Selected positive samples underwent sequencing analysis targeting the second hypervariable region of the *G* gene using next-generation sequencing.

**Results:**

The RSV positivity rate was 28.02% (438/1563 samples), and prevalence was highest in children aged < 1 year (43.84%; 192/438). Subtype RSV-A accounted for 53.42% (234/438) of cases, RSV-B for 45.89% (201/438), and there was coinfection in 0.68% (3/438). Both subtypes cocirculated and peaked during August–September in each year of the study. Phylogenetic analysis showed that RSV-A samples belonged to the ON1 genotype, which has three subgenotypes: ON1.1, ON1.2 and ON1.3. However, we did not find the 72-nucleotide duplication in the second hypervariable region of the *G* gene, a characteristic of genotype ON1, in any RSV-A samples. RSV-B samples belonged to genotype BA9.

**Discussion:**

Our results provide additional molecular characterization of RSV infections in Viet Nam. Specially, our study is the first to report the absence of the 72-nucleotide duplication in the *G* gene of RSV-A genotype ON1 in Viet Nam, which may help in understanding the genetic evolution of RSV and be useful for vaccine development in the future.

Human respiratory syncytial virus (RSV) is one of the most common causes of severe acute respiratory infection (SARI) among children worldwide. Most children have at least one episode of RSV infection by the age of 2 years, and 5% of these cases require hospitalization. (*1*) RSV belongs to the recently defined family *Pneumoviridae*, genus *Orthopneumovirus*, and consists of a single-stranded, negative-sense RNA genome packaged in a lipid envelope. It has about 15.2 kb and contains 10 genes encoding 11 viral proteins. The external glycoproteins F and G are the two primary antigens for viral attachment and make a syncytial form. (*2*) The G protein, especially in the second hypervariable region (HVR), is highly genetically diverse and under selection pressure, and thus is used for the molecular characterization of RSV strains. (*3*)

RSV is classified into two subgroups – RSV-A and RSV-B – based on the difference in the second HVR of the *G* gene. Currently, RSV-A is divided into 12 genotypes (GA1–7, SAA1, NA1–2, ON1–2), and RSV-B is divided into 32 genotypes (GB1–5, BA1–14, SAB1–4, URU1–2, NZB1–2, BA-CCA, BA-CCB, BA-C, CBB, CB1). (*4*) In many countries, the predominant genotypes are RSV-A ON1, with a 72-nucleotide duplication in the HVR of the *G* gene, and RSV-B BA, with a 60-nucleotide duplication in the HVR of the *G* gene. (*5*)

RSV infections range in severity from common cold symptoms to severe, acute symptoms, including pneumonia or bronchiolitis requiring hospitalization. It has been estimated that 2.8–4.3 million children with RSV infection are admitted to hospital each year worldwide, and approximately 66 000–199 000 children aged < 5 years die, particularly in developing countries. (*6*) Human RSV has a seasonal epidemic pattern similar to that of influenza. In Europe, northern Asia and North America, the seasonal RSV epidemic occurs in the winter and early spring months. (*7*) By contrast, in tropical countries, RSV cases are seen year-round and peak during the rainy season or in the months with the lowest temperatures and highest rainfall. (*8*)

Research on RSV in Viet Nam has been limited, mostly completed before 2016 and focused on coinfections with other respiratory pathogens. Surveillance data show that RSV usually occurs in the winter, when the temperature in the northern region is lowest. (*9*) However, that research spanned only 1–2 years, and the majority of research was conducted in central and southern Viet Nam.

The objective of this study was to analyse the circulation of RSV in northern Viet Nam during 2017–2020 and to investigate the genetic variability of the second HVR of the *G* gene to characterize the evolution of RSV in Viet Nam.

## Methods

### Sample collection

The study was conducted from January 2017 to December 2020 in a paediatric hospital in Hanoi and a general hospital in Quang Ninh province. Children aged < 16 years who were admitted with SARI were enrolled. The definition of SARI followed World Health Organization guidelines: fever of ≥ 38 °C, cough, onset of symptoms within the past 10 days and illness requiring hospitalization. (*9*) Written consent for study enrolment was obtained from children’s parents or legal guardians. Demographic data were recorded on a surveillance questionnaire.

Specimens of nasopharyngeal aspirate and nose–throat swabs were collected from children with SARI 1 day after hospital admission. A maximum of 10 new children were selected weekly for specimen collection in each hospital. Specimens were stored in a viral transport medium at −20 °C until they could be transferred to the National Institute of Hygiene and Epidemiology for testing. All samples were collected for routine surveillance of respiratory viruses under Decision No. 4608/QD-BYT of the Ministry of Health, Viet Nam, which governs ethical conduct during research, among other areas.

### Screening and subtyping

Viral RNA was extracted directly from the specimens using the QIAamp Viral RNA Mini Kit (QIAGEN, Hilden, Germany), according to the manufacturer’s instructions. All samples were screened for RSV using real-time reverse transcription–polymerase chain reaction (RT–PCR), with primer and probe sequences following the protocol of the United States Centers for Disease Control and Prevention. (*10*) Subtyping of the RSV strains was achieved using publicly available primers and probes based on the highly conserved genomic regions on the *N* gene for the subgroups RSV-A and RSV-B. (*11*) SuperScript III Platinum One-Step qRT–PCR reagent (Invitrogen, ThermoFisher Scientific, Waltham, MA, USA) was used with a thermocycler appropriate to each protocol.

### Sequencing the second highly variable region of the *G* gene

Specimens that were positive for RSV were selected for sequencing by subtype, age, sex, collection year and hospital. Viral RNA for screening and subtyping was transcribed to copy DNA using the SuperScript IV First-Strand synthesis system (Invitrogen). Conventional PCR targeting of the second HVR was performed using Platinum SuperFi II Green PCR Master Mix (Invitrogen) and primers as described by Hibino et al. (*12*)

PCR products were purified with ExoSAP-IT PCR Product Cleanup Reagent (ThermoFisher Scientific) and diluted to 0.2 ng/µL. The library used for sequencing followed the protocol of the Nextera XT DNA Library Preparation Kit (Illumina, San Diego, CA, USA). The final concentration of the library was 60 pM for elution into the cartridge of the Illumina iSeq 100 System.

### Phylogenetic tree and genotyping analyses

Sequencing data were primarily analysed using CLC Genomics WorkBench v. 11.0 (QIAGEN). First, the FASTQ file was quality controlled, and then the low-quality sequences and the noise in the 3′ and 5′ terminals were trimmed. After trimming, all reads were mapped with representative subtype references to create final consensus. The nucleotide and amino acid substitutions of the second HVR of all RSV-A and RSV-B strains in this study were compared with, respectively, those of the prototype lineage ON1 (GenBank accession number JN257693) and BA9 (GenBank accession number AY333364).

Phylogenetic trees of the *G* gene’s second HVR were generated using maximum likelihood estimation with MEGA (Molecular Evolutionary Genetics Analysis) v. 10 software (https://www.megasoftware.net/). Bootstrap probabilities were calculated with 1000 replications to evaluate confidence estimates. Genotypes were assigned with a 72-nucleotide duplication in RSV-A and

60-nucleotide duplication in RSV-B in the second HVR, as in ON1 and BA, respectively. Known genotype sequences from other countries were used as references for more accuracy. Subgenotypes for RSV-A and genotypes for RSV-B were identified using reference sequences from, respectively, Myanmar and China, Taiwan (China). (*5*, *6*) All Vietnamese RSV sequences were submitted to the Global Initiative on Sharing All Influenza Data (GISAID)database (accession numbers EPI_ISL_16051837 to EPI_ISL_16051941).

### Statistical analyses

Patients’ information and test results were imported into Filemaker Pro software (Claris, Apple, Cupertino, CA, USA). These data were stored and analysed by the National Institute of Hygiene and Epidemiology. Stata v. 14 (StataCorp, College Station, TX, USA) and Microsoft Excel software (Microsoft, Redmond, WA, USA) were used for testing epidemiological characteristics and graphing RSV surveillance data. *P*-values < 0.05 were considered statistically significant.

## Results

### Circulation of RSV

During the study period (2017–2020), 1563 specimens were collected and 438 (28.02%) tested positive for RSV. The number of samples in each year was not similar, with the highest number in 2017 (512) and the lowest in 2020 (263). The RSV positivity rate in 2017 was much higher than the rate in other years, at 36.13% (**Table 1**). The difference in the rate of RSV screening each year was statistically significant (*P* < 0.05).

**Table 1:** Number of samples tested for respiratory syncytial virus (RSV), positivity rate, subtype prevalence and distribution of RSV-positive cases by age group and sex in paediatric cases of severe acute respiratory infection, two sentinel sites in northern Viet Nam, 2017–2020

Characteristic	Year^a^				
	2017	2018	2019	2020	2017–2020
**No. of samples tested**					
Total	512/1563 (32.76)	420/1563 (26.87)	368/1563 (23.54)	263/1563 (16.83)	1563/1563 (100)
RSV+	185/512 (36.13)	109/420 (25.95)	77/368 (20.92)	66/263 (25.10)	438/1563 (28.02)
RSV−	327/512 (63.87)	311/420 (74.05)	291/368 (79.08)	197/263 (74.90)	1125/1563 (71.98)
**Subtype**					
No. of subtyped samples	185/185 (100)	109/109 (100)	77/77 (100)	66/66 (100)	438/438 (100)
RSV-A	99/185 (53.51)	59/109 (54.13)	30/77 (38.96)	45/66 (68.18)	234/438 (53.42)
RSV-B	83/185 (44.86)	50/109 (45.87)	47/77 (60.26)	21/66 (31.82)	201/438 (45.89)
RSV-A and RSV-B	3/185 (1.62)	0/109 (0)	0/77 (0)	0/66 (0)	3/438 (0.68)
**Age (years) of RSV+ cases**					
< 1	118/185 (63.78)	54/109 (49.54)	9/77 (11.54)	11/66 (16.67)	192/438 (43.84)
1 to < 2	44/185 (23.78)	40/109 (36.70)	13/77 (16.67)	23/66 (34.85)	120/438 (27.40)
2 to < 5	16/185 (8.65)	13/109 (11.93)	26/77 (33.33)	25/66 (37.88)	80/438 (18.26)
≥ 5	7/185 (3.78)	2/109 (1.83)	29/77 (37.66)	7/66 (10.61)	46/438 (10.50)
**Sex of RSV+ cases**					
Male	117/185 (63.24)	64/109 (58.72)	40/77 (51.28)	36/66 (54.55)	257/438 (58.67)
Female	68/185 (36.76)	45/109 (41.28)	37/77 (48.05)	30/66 (45.45)	181/438 (41.32)

RSV: respiratory syncytial virus.

^a^ Values are numbers (%).

The highest numbers of positive cases occurred in children aged < 1 year and those aged 1 to < 2 years. The rate of RSV positivity in the youngest age group was about four times higher than that in the group aged > 5 years (*P* < 0.05). Although RSV positivity in males was higher than in females, this difference was not significant (**Table 1**).

### Prevalence of RSV subtypes and genotypes

Both RSV subtypes cocirculated between 2017 and 2020. During 2017 and 2018, there were similar proportions of each subtype, at around 50%. Conversely, during 2019 and 2020, RSV-A and RSV-B circulated alternately, with RSV-B predominant in 2019 and RSV-A in 2020. In 2017, 3/185 patients (1.62%) were coinfected with both subtypes (**Table 1**). During the study period, there was no significant difference in the rate of infection with RSV-A or RSV-B.

**Fig. 1:**
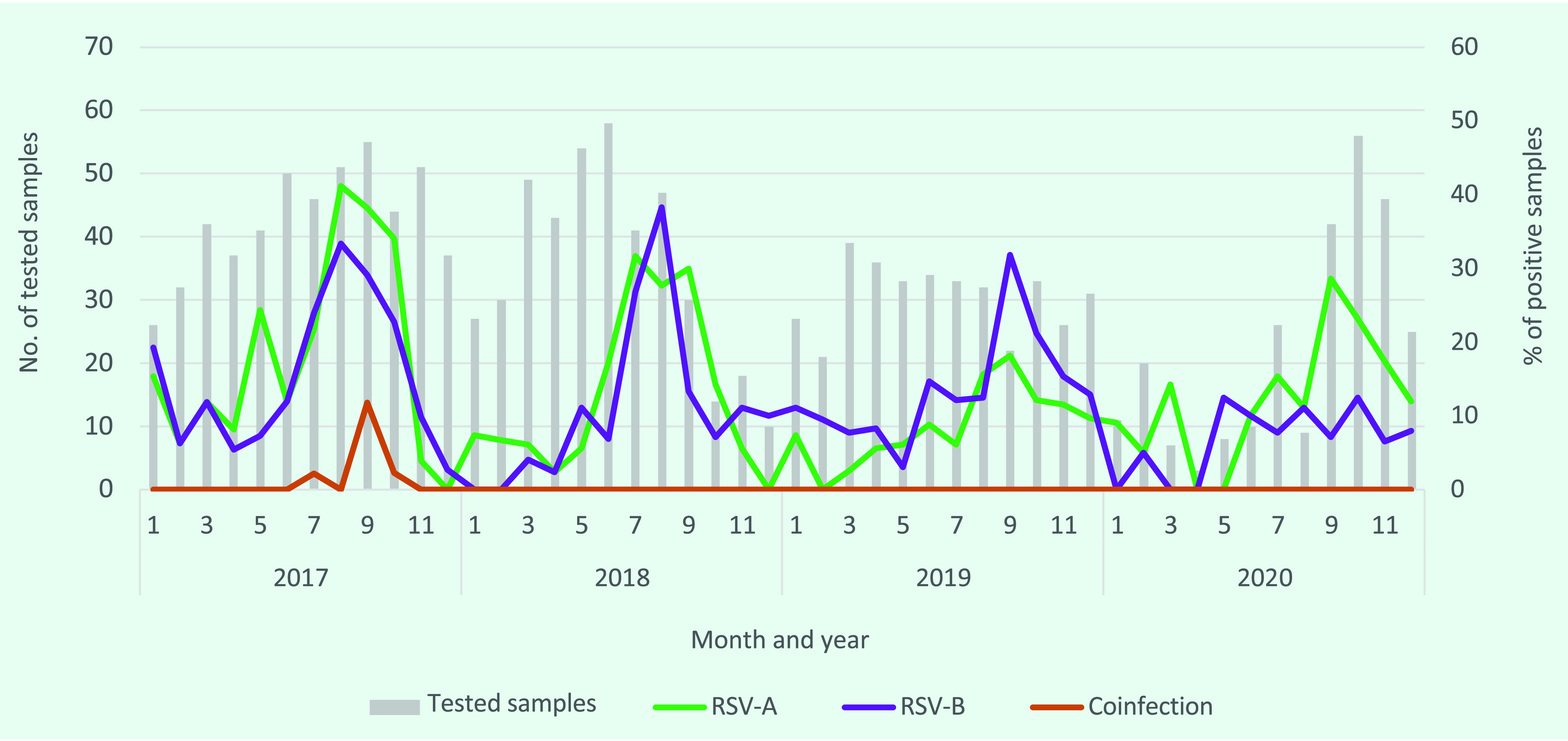
Number of samples tested and proportion positive for respiratory syncytial virus (RSV) subtypes A and B and coinfection with RSV-A and RSV-B in paediatric cases of severe acute respiratory infection, two sentinel sites in northern Viet Nam, 2017–2020

The frequency of RSV infection increased beginning every July (month 7), peaking at approximately 40% of tested samples in August–September (months 8 and 9), then falling for the rest of the year (**Fig. 1**). In 2017 and 2018, there was cocirculation of both subtypes of RSV, with peaks in August of both years. A similar time trend was seen in 2019, although RSV-B predominantly circulated. In contrast, in 2020, infection with RSV-A accounted for the highest proportion of cases, while the rate of infection with RSV-B was almost unchanged during the peak period.

**Fig. 2:**
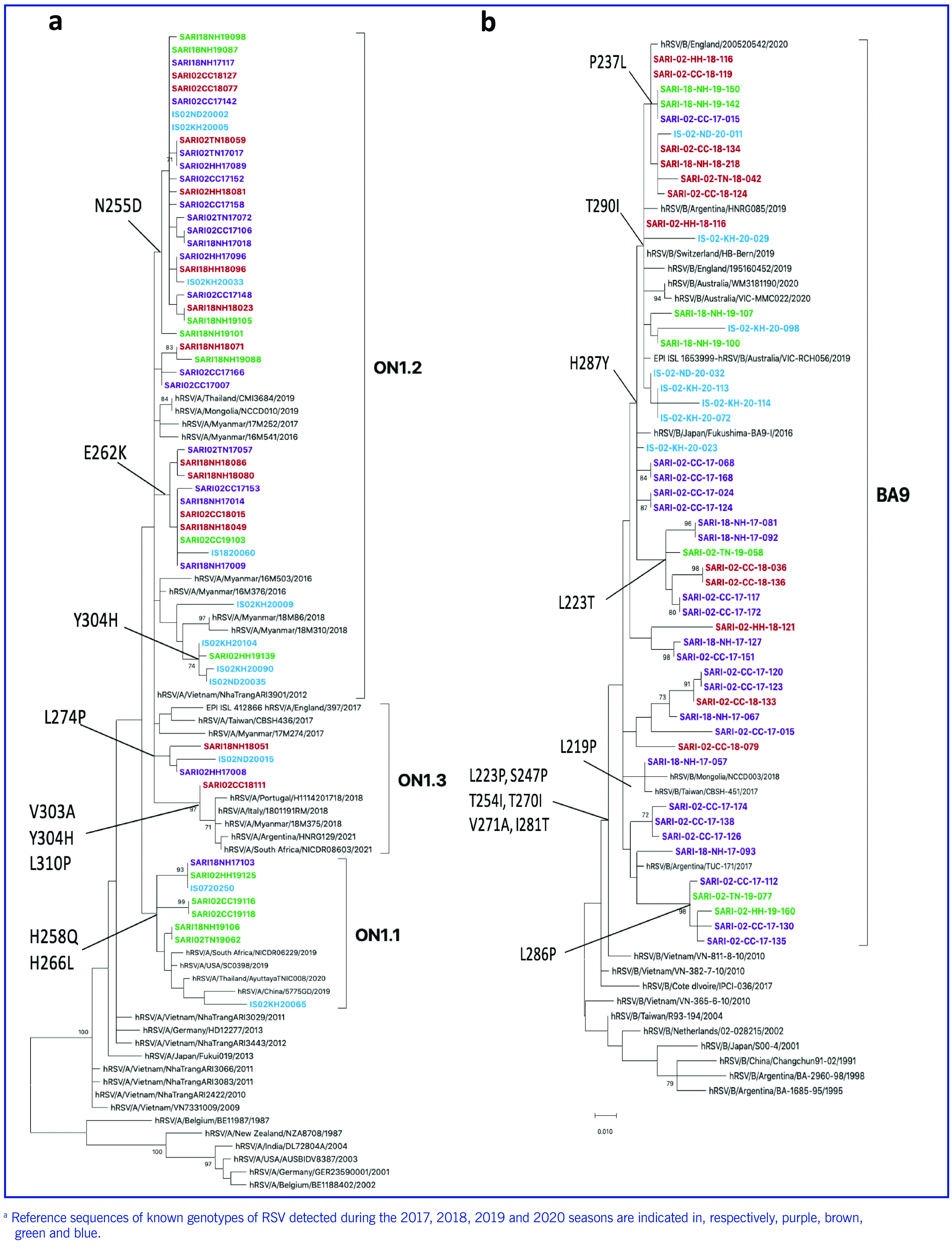
Phylogenetic analysis of the second hypervariable region of the G gene in Vietnamese respiratory syncytial virus (RSV) subtypes (a) A and (b) B from paediatric cases of severe acute respiratory infection, two sentinel sites in northern Viet Nam, 2017–2020a

**Fig. 3:**
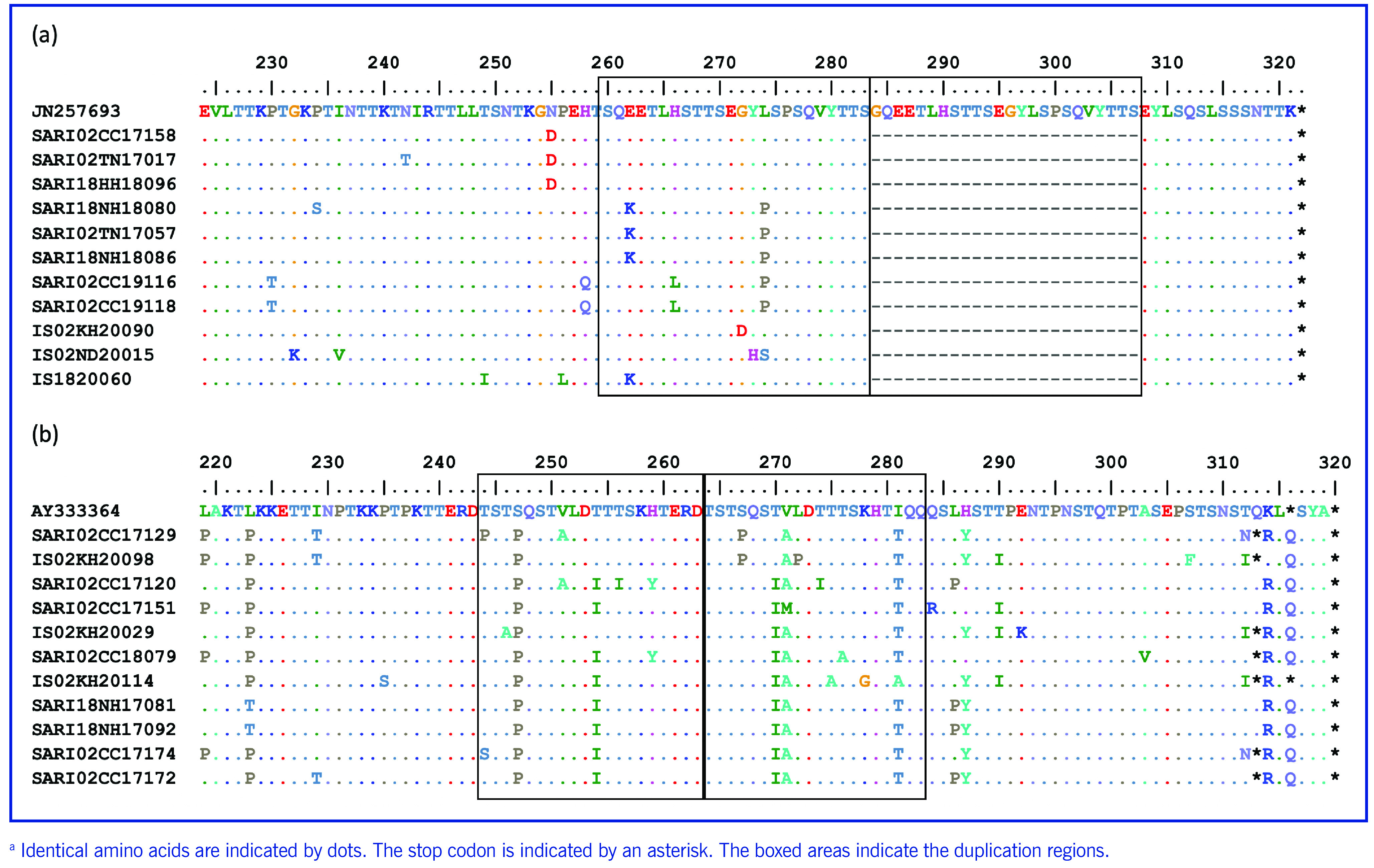
Alignment of deduced amino acids of representative samples of Vietnamese respiratory syncytial virus (RSV) (a) subtype A genotype ON1 compared with prototype lineage JN257693 (GenBank accession number) and (b) RSV-B genotype BA9 compared with prototype lineage AY333364 from paediatric cases of severe acute respiratory infection, two sentinel sites in northern Viet Nam, 2017–2020a

Altogether, 105 sequences of the second HVR of the *G* gene were obtained. Phylogenetic analysis revealed that all Vietnamese RSV-A strains (*n* = 55) belonged to the ON1 genotype (**Fig. 2a**) and clustered with RSV sequences from Italy, Myanmar, China, Taiwan (China) and Thailand. ON1 samples identified in Viet Nam during the 2017–2020 seasons were located in three lineages: ON1.1, ON1.2 and ON1.3. Although the phylogenetic tree characterized Vietnamese RSV-A as belonging to genotype ON1, these sequences did not have the 72-nucleotide duplication between amino acids 284 and 307 (GQEETLHSTTSEGYLSPSQVYTTS) (**Fig. 3a**).

[**insert** Figure 1]

[**insert** Figure 2]

Most of the Vietnamese sequences had the amino acid substitution N255D (24/55) or E262K (10/55), both of which are characteristic of strains belonging to subgenotype ON1.2. Moreover, the specimens in subgenotype ON1.1 shared the same substitutions: H258Q and H266L. Only one substitution, Y304H, was seen in several strains in both the ON1.1 and ON1.2 lineages. All RSV-A specimens in subgroup ON1.3 had one major amino acid substitution: L274P.

The results of the phylogenetic tree for the 50 RSV-B samples showed that they belonged to the BA9 genotype (**Fig. 2b**). All Vietnamese sequences had the insertion of a 60-nucleotide duplication, which means that 20 amino acids (TERDTSTSQSTVLDTTTSKH) were inserted in positions 260–279 (**Fig. 3b**). The Vietnamese BA9 viruses had two different G protein lengths, of 312 and 319 amino acids. All Vietnamese RSV-B sequences were in the same group as sequences from Argentina, England, Mongolia and China, Taiwan (China) and shared six substitutions (L223P, S247P, T254I, T2701, V271A, I281T).

[**insert** Figure 3]

## Discussion

In this study, we investigated the circulation of RSV and its genotypic variations in the second HVR during 2017–2020 in northern Viet Nam. The RSV prevalence in children with SARI was 28.02% during these 4 years. This rate was similar to that in Myanmar (24.5%), (*6*) but higher than that in China (16%) (*13*) and Thailand (13.2%), (*14*) and lower than in Brazil (56%). (*1*) The rate of RSV infection in children in central Viet Nam during 2007–2012 was 26.8%, (*15*) whereas SARI specimens from five representative locations in Viet Nam during 2012–2016 showed that the rate of RSV positivity was 22.8%. (*16*) The RSV positivity rate among paediatric patients in Viet Nam seemed to fluctuate at around 20–30%, depending on the study location and time of sample collection.

These studies showed diversity in the rate of RSV positivity by age. In our study, the difference in RSV positivity was statistically significant between children < 2 years and those > 5 years. This means that children < 2 years have a higher risk of infection than those > 5 years (*P* < 0.05). This trend was also seen in other research, where the strongest risk factor for RSV infection was age. (*13*)

Some previous studies have found no correlation between subtypes and circulation or disease severity, whereas others have shown that RSV-A was more common and virulent than RSV-B. (*1*) In Viet Nam, there has not been much reporting on the surveillance of RSV subtypes. During our study, RSV-A and RSV-B circulated in parallel during 2017–2020, and cocirculated during the first 2 years. In one analysis of the Netherlands, New Zealand, Portugal and South Africa, out of 24 seasons during 2010–2019 with RSV subtype data available, RSV-A showed at least 60% dominance in 10 seasons and RSV-B in eight seasons, while neither reached 60% in the remaining six seasons. (*17*) In 2017, RSV-A predominated in Argentina (91% of samples) and the United Kingdom of Great Britain and Northern Ireland (62.5%), while RSV-B predominated in Australia (75%), India (98%), South Africa (64.4%) and Thailand (57%). (*8*) These results show no clear patterns in dominant subtype by season or geography, and highlight the need for more countries to collect data on subtypes to better understand their global circulation.

The coronavirus disease (COVID-19) pandemic not only led to a worldwide health crisis but also had a great impact on the circulation of other respiratory viruses, including RSV. According to a report from New Zealand, the number of paediatric hospitalizations for SARI and the number of single RSV or influenza infections decreased significantly during the pandemic. (*18*) In Thailand, the seasonal RSV peak was delayed by 2 months in 2020, (*19*) and in the Republic of Korea, the rates of RSV and influenza positivity were close to zero in the first half of the 2020–2021 season, their seasonal peak. (*20*) However, in the 2020 season in Viet Nam, the rate of RSV positivity was 25.1%, with RSV-A predominant and peaking in September. By comparison, morbidity from RSV-B was stable at around 10%. In 2020, the prevalence of RSV did not change much despite the COVID-19 pandemic. The Government of Viet Nam had implemented strict border control measures to limit the spread of COVID-19, resulting in only 1551 confirmed cases and 35 deaths reported nationally by late January 2021, most of them in southern Viet Nam. (*21*) Business operations, manufacturing, travel and study were not greatly affected. Children attended school on-site for the entire academic year, except for 2 weeks of lockdown in April 2020. Although the number of samples tested for RSV was low during the first half of 2020 due to the public’s avoidance of hospitals caring for COVID-19 patients, the percentage of positive samples was largely unchanged throughout the year (**Fig. 1**), suggesting that the COVID-19 pandemic may have only weakly affected RSV prevalence in northern Viet Nam. However, our study period included only the first year of the pandemic; RSV data from subsequent years of the pandemic should be analysed as well.

The molecular epidemiological testing conducted in this study showed that the predominant RSV-A subtype was associated with the ON1 genotype, which was classified based on the phylogenetic tree. The ON1 genotype was first identified in Canada in 2010, with a 72-nucleotide insertion in the second HVR of the *G* gene. (*6*) This genotype subsequently spread rapidly across the world. However, in this study, the strains that lost the 72-nucleotide duplication in the second HVR of the *G* gene were still classified as RSV-A ON1. These results differ from most previous research, (*3*, *4*) which found that the ON1 strain had a duplication region in the second HVR of the *G* gene.

Analysis of worldwide nucleotide sequences of the second HVR and the complete *G* gene have suggested a high similarity between the ON1 and NA1 genotypes (p-distance = 0.029). (*22*) Therefore, phylogenetic tree analysis indicated that ON1 does not constitute a separate genotype from NA1. ON1 was within the NA1 genotype despite showing distinct genetic characteristics, including the 72-nucleotide duplication. The lost duplication region in our RSV sequences was identified as belonging to the ON1 genotype, suggesting that genotype designation should be based on a systematic analysis of the phylogenetic tree regardless of the presence of a duplication insertion.

Compared with Vietnamese RSV sequences in the past, the ON1 genotype had a duplication region in strains in 2012 (*23*) and 2013–2015. (*16*) These sequences were also referenced in our phylogenetic tree. Moreover, RSV strains in 2013–2015 were collected from five facilities representing the three regions of Viet Nam, while our sequences were only from northern Viet Nam. This suggests that ON1 strains in northern Viet Nam during 2017–2020 differed from those circulating during 2013–2015 in terms of the 72-nucleotide duplication. Tabatabai et al. mentioned the deletion of the 72-nucleotide duplication in genotype ON1 in their research on patients with haematological disease, (*24*) suggesting that ON1 strains without the duplication were not Vietnamese domestic strains of RSV, but rather they might be the consequence of imported infections.

The RSV-B BA genotype was defined by the 60-nucleotide duplication region in the second HVR of the *G* gene. RSV-B BA was first reported in 1999, and since 2015, BA9 has been the predominant genotype worldwide. (*3*) Research in Viet Nam from 2010 to 2020 has shown that BA9 circulates more commonly than other subgenotypes. (*16*, *25*) Additionally, the Vietnamese BA9 viruses had two different G protein lengths of 312 and 319 amino acids. During 2010–2011, this was reported not only in Viet Nam but also in other countries. (*25*, *26*)

This study has several limitations. First, virus samples were collected from children who were hospitalized with SARI. The prevalence of RSV in patients in the community was not analysed. Therefore, the results may not be representative of all RSV circulating in Viet Nam. In addition, we surveyed two sentinel hospitals that belong to Viet Nam’s national influenza surveillance system, under the direction of the Ministry of Health. Although the number of specimens collected was enough for calculations, the two hospitals may not fully represent northern Viet Nam. Last, we primarily sequenced the second HVR of the *G* gene in RSV genomes. Several significant results were identified in the classification of the RSV-A subtype and in amino acid substitutions in RSV-B. Therefore, an extension of genomic sequencing is necessary to further analyse the molecular characteristics of RSV in northern Viet Nam.

The 28.02% RSV positivity rate among paediatric SARI cases in the present study was similar to rates found in Viet Nam previously. Children < 1 year had the highest positivity rate. RSV circulated year-round and reached a peak of nearly 40% sample positivity during August–September every year. Both RSV-A and RSV-B were seen during 2017–2020, with RSV-B predominant in 2019 and RSV-A predominant in 2020. RSV-A sequences belonged to genotype ON1 in three lineages (ON1.1, ON1.2, ON1.3), and RSV-B sequences belonged to genotype BA9. Although all Vietnamese RSV-A samples in this study were genotype ON1, they did not have the 72-nucleotide duplication in the second HVR of the *G* gene, which differentiates them from findings in previous research in Viet Nam. This is the first report of the new ON1 genotype without the duplication in Viet Nam.

## References

[1] Vianna LA, Siqueira MM, Volpini LPB, Louro ID, Resende PC. Seasonality, molecular epidemiology, and virulence of respiratory syncytial virus (RSV): a perspective into the Brazilian Influenza Surveillance Program. PLoS One. 2021 May 18;16(5):e0251361. 10.1371/journal.pone.0251361 34003843 PMC8130917

[2] Pandya MC, Callahan SM, Savchenko KG, Stobart CC. A contemporary view of respiratory syncytial virus (RSV) biology and strain-specific differences. Pathogens. 2019 May 21;8(2):67. 10.3390/pathogens8020067 31117229 PMC6631838

[3] Yu JM, Fu YH, Peng XL, Zheng YP, He JS. Genetic diversity and molecular evolution of human respiratory syncytial virus A and B. Sci Rep. 2021 Jun 21;11(1):12941. 10.1038/s41598-021-92435-1 34155268 PMC8217232

[4] Yun KW, Choi EH, Lee HJ. Molecular epidemiology of respiratory syncytial virus for 28 consecutive seasons (1990-2018) and genetic variability of the duplication region in the G gene of genotypes ON1 and BA in South Korea. Arch Virol. 2020 May;165(5):1069–77. 10.1007/s00705-020-04580-z 32144544

[5] Lee CY, Fang YP, Wang LC, Chou TY, Liu HF. Genetic diversity and molecular epidemiology of circulating respiratory syncytial virus in central Taiwan, 2008–2017. Viruses. 2021 Dec 24;14(1):32. 10.3390/v14010032 35062237 PMC8777914

[6] Phyu WW, Htwe KTZ, Saito R, Kyaw Y, Lin N, Dapat C, et al. Evolutionary analysis of human respiratory syncytial virus collected in Myanmar between 2015 and 2018. Infect Genet Evol. 2021 Sep;93:104927. 10.1016/j.meegid.2021.104927 34020068

[7] Tin Tin Htar M, Yerramalla MS, Moïsi JC, Swerdlow DL. The burden of respiratory syncytial virus in adults: a systematic review and meta-analysis. Epidemiol Infect. 2020 Feb 13;148:e48. 10.1017/S0950268820000400 32052719 PMC7078512

[8] Chadha M, Hirve S, Bancej C, Barr I, Baumeister E, Caetano B, et al.; WHO RSV Surveillance Group. Human respiratory syncytial virus and influenza seasonality patterns-Early findings from the WHO global respiratory syncytial virus surveillance. Influenza Other Respir Viruses. 2020 Nov;14(6):638–46. 10.1111/irv.12726 32163226 PMC7578323

[9] Hoang VMP, Le TT, Nguyen VS, Ung THT, Vuong DC, Pham TH, et al. [Respiratory virus infection caused severe acute respiratory infection in children under 5 years old admitted to the national paediatric hospital, 2016]. Vietnam J Prevent Med. 2017;27(8):255–61 (in Vietnamese). Available from: http://www.tapchiyhocduphong.vn/tap-chi-y-hoc-du-phong/2017/08/mot-so-can-nguyen-vi-rut-ho-hap-gay-nhiem-trung-duong-ho-hap-cap-tinh-nang-o-ben-o81E20652.html, accessed 10 October 2017.

[10] Real-time RT-PCR assays for non-influenza respiratory viruses. Atlanta (GA): Centers for Disease Control and Prevention; 2015. pp. 1–16.

[11] Do LA, van Doorn HR, Bryant JE, Nghiem MN, Nguyen Van VC, Vo CK, et al. A sensitive real-time PCR for detection and subgrouping of human respiratory syncytial virus. J Virol Methods. 2012 Jan;179(1):250–5. 10.1016/j.jviromet.2011.11.012 22119628 PMC3405522

[12] Hibino A, Saito R, Taniguchi K, Zaraket H, Shobugawa Y, Matsui T, et al.; Japanese HRSV Collaborative Study Group. Molecular epidemiology of human respiratory syncytial virus among children in Japan during three seasons and hospitalization risk of genotype ON1. PLoS One. 2018 Jan 29;13(1):e0192085. 10.1371/journal.pone.0192085 29377949 PMC5788364

[13] Suleiman-Martos N, Caballero-Vázquez A, Gómez-Urquiza JL, Albendín-García L, Romero-Béjar JL, Cañadas-De la Fuente GA. Prevalence and risk factors of respiratory syncytial virus in children under 5 years of age in the WHO European Region: a systematic review and meta-analysis. J Pers Med. 2021 May 15;11(5):416. 10.3390/jpm11050416 34063453 PMC8155861

[14] Thongpan I, Vongpunsawad S, Poovorawan Y. Respiratory syncytial virus infection trend is associated with meteorological factors. Sci Rep. 2020 Jul 2;10(1):10931. 10.1038/s41598-020-67969-5 32616819 PMC7331681

[15] Althouse BM, Flasche S, Minh LN, Thiem VD, Hashizume M, Ariyoshi K, et al. Seasonality of respiratory viruses causing hospitalizations for acute respiratory infections in children in Nha Trang, Vietnam. Int J Infect Dis. 2018 Oct;75:18–25. 10.1016/j.ijid.2018.08.001 30118916 PMC7110808

[16] Lu L, Robertson G, Ashworth J, Pham Hong A, Shi T, Ivens A, et al. Epidemiology and phylogenetic analysis of viral respiratory infections in Vietnam. Front Microbiol. 2020 May 15;11:833. 10.3389/fmicb.2020.00833 32499763 PMC7242649

[17] Staadegaard L, Caini S, Wangchuk S, Thapa B, de Almeida WAF, de Carvalho FC, et al. Defining the seasonality of respiratory syncytial virus around the world: National and subnational surveillance data from 12 countries. Influenza Other Respir Viruses. 2021 Nov;15(6):732–41. 10.1111/irv.12885 34255934 PMC8542954

[18] Trenholme A, Webb R, Lawrence S, Arrol S, Taylor S, Ameratunga S, et al. COVID-19 and infant hospitalizations for seasonal respiratory virus infections, New Zealand, 2020. Emerg Infect Dis. 2021 Feb;27(2):641–3. 10.3201/eid2702.204041 33263515 PMC7853573

[19] Thongpan I, Vichaiwattana P, Vongpunsawad S, Poovorawan Y. Upsurge of human rhinovirus infection followed by a delayed seasonal respiratory syncytial virus infection in Thai children during the coronavirus pandemic. Influenza Other Respir Viruses. 2021 Nov;15(6):711–20. 10.1111/irv.12893 34350701 PMC8542963

[20] Kim JH, Roh YH, Ahn JG, Kim MY, Huh K, Jung J, et al. Respiratory syncytial virus and influenza epidemics disappearance in Korea during the 2020-2021 season of COVID-19. Int J Infect Dis. 2021 Sep;110:29–35. 10.1016/j.ijid.2021.07.005 34245886

[21] Hoang VT, Pham TD, Nguyen QT, Nguyen DC, Nguyen DT, Nguyen TB, et al. Seroprevalence of SARS-CoV-2 among high-density communities and hyper-endemicity of COVID-19 in Vietnam. Trop Med Int Health. 2022 May;27(5):515–21. 10.1111/tmi.13744 35303386 PMC9115418

[22] Muñoz-Escalante JC, Comas-García A, Bernal-Silva S, Robles-Espinoza CD, Gómez-Leal G, Noyola DE. Respiratory syncytial virus A genotype classification based on systematic intergenotypic and intragenotypic sequence analysis. Sci Rep. 2019 Dec 27;9(1):20097. 10.1038/s41598-019-56552-2 31882808 PMC6934736

[23] Yoshihara K, Le MN, Nagasawa K, Tsukagoshi H, Nguyen HA, Toizumi M, et al. Molecular evolution of respiratory syncytial virus subgroup A genotype NA1 and ON1 attachment glycoprotein (G) gene in central Vietnam. Infect Genet Evol. 2016 Nov;45:437–46. 10.1016/j.meegid.2016.10.010 27746294

[24] Tabatabai J, Thielen A, Lehners N, Daeumer M, Schnitzler P. Respiratory syncytial virus A in haematological patients with prolonged shedding: Premature stop codons and deletion of the genotype ON1 72-nucleotide-duplication in the attachment G gene. J Clin Virol. 2018 Jan;98:10–7. 10.1016/j.jcv.2017.11.003 29175230

[25] Tran DN, Pham TMH, Ha MT, Tran TT, Dang TK, Yoshida LM, et al. Molecular epidemiology and disease severity of human respiratory syncytial virus in Vietnam. PLoS One. 2013;8(1):e45436. 10.1371/journal.pone.0045436 23349659 PMC3551923

[26] Haider MSH, Khan WH, Deeba F, Ali S, Ahmed A, Naqvi IH, et al. BA9 lineage of respiratory syncytial virus from across the globe and its evolutionary dynamics. PLoS One. 2018 Apr 25;13(4):e0193525. 10.1371/journal.pone.0193525 29694383 PMC5919079

